# Reviewers acknowledgement

**DOI:** 10.1002/2211-5463.13329

**Published:** 2021-12-01

The editors of *FEBS Open Bio* would like to thank all those who have given their time and expertise to review articles submitted for publication in Volume 11. Although *FEBS Open Bio* does not seek to judge the importance of submissions, our reviewers carefully scrutinize the experimental design and results of all papers and challenge authors about their conclusions.

The names of these reviewers are listed below; we apologize if we have inadvertently omitted anyone.

Tarek Abbas

Enass Abdel‐hameed

Chérine Abou‐Faycal

Jason Acker

Loranne Agius

Emad Ahmed

Joong‐Hoon Ahn

Wado Akamatsu

Satoshi Akunama

Ayodele Alaiya

Arshad Ali

Malcolm Alison

David S. Allan

Chiara Allegretti

Marco G. Alves

Valentin Ambroise

Emmanuel Ampofo

Kanae Ando

Sylvia Andrzejewski

Christophe Anjard

Siddhesh Aras

Patrick Arbuthnot

Isaac Armendáriz‐Castillo

Paolo Arosio

Juan José Arredondo

Venkatesan Arul

Kazuhito Asano

Avraham Ashkenazi

Essam Assali

Ianire Astobiza

Nese Atabey

Riccardo Autelli

Angelo Azzi

Monika Baj‐Krzyworzeka

Oliver Bajt

Marica Bakovic

Ovidiu Balacescu

Miroslav Balaz

Pedro Ballester

Lawrence Banks

Janos Barna

Maureen Barr

Reetobrata Basu

Daniel Batlle

Maurizio Battino

Maria Eduarda Battistella

Daniel J. Becker

Alexandre Benedetto

Alfonso Benitez‐Paez

Ivan A. Berg

Christina Bergonzo

Isabel Bermudez

Valeria Bertagnolo

Aditi Bhargava

Arindam Bhattacharjee

Farzana Bhuyan

Fabrizio Bianchi

Jana Biermann

Saikat Biswas

Francisco Blanco

Bogdan Bochenek

Sreedhar Bodiga

Mirian Aparecida Boim

Mariela Bollati‐Fogolin

Marielle Boonen

Usa Boonyuen

Francesca Bosisio

Stephanie Marie Bozonet

Klaudia Brix

Leyre Brizuela

Federica Brugnoli

Paola Bruni

Viljemka Bučević Popović

Beáta Bugyi

Philipe Bulet

Leif Bülow

Michal Burdukiewicz

Pedro Buzon‐Rodriguez

Jonas Bystrom

Filipe Cabreiro

Javier Caceres

Lukas Cajanek

Jesús Campos‐García

Reinaldo Campos‐Vargas

Ihosvany Camps

Liu Cao

Silvia Capellino

Giuseppina Caretti

Anthony Carlos

Bruno M. Carneiro

Ingrid Carvacho

Miguel A. Casado‐Combreras

Sara Castiglioni

João Cavalcanti

David L. Cedeño

Richard A. Cerione

Igor Cesarino

Jobichen Chacko

Parin Chaivisuthangkura

Ushati Das Chakravarty

Hsueh‐Wei Chang

Mark Chappell

Kausik Chattopadhyay

Lee‐Young Chau

Qi Chen

Yu Chen

Min Chen

Jinbo Cheng

Jorge O. Chiapella

Ozioma Chioma

Esther S. Choi

Yu‐Ching Chou

Goh Eun Chung

Valerio Ciccone

Patricia Coltri

James R. Connor

F. Xabier Contreras

Lucia Coppo

Paulo Correia‐de‐Sá

Federica Cossu

Edward J. Crane

Ute Curth

Agnes Czibula

Zsolt Czimmerer

Jasmina Damnjanovic

Kirsty M. Danielson

Tatyana Danyukova

Jean Luc Darlix

Mads Daugaard

Alberto Davalos

Albert Davis

Regina Day

Juan Carlos de la Torre

Michael Dean

Vishwa Deepak

Constantinos A. Alexandros Demopoulos

Liesbeth Demuyser

Yuru Deng

Tuba Denkçeken

Jayne Dennis

Tom Desmet

Satish Devadas

Kishor Devalaraja‐Narashimha

Irene Díaz‐Moreno

Antonio J. Diaz‐Quintana

Price Dickson

Bauke W. Dijkstra

Vittoria Disciglio

Francesco Dituri

Jose Donato

Fabian Dorninger

Leonardo dos Reis Silveira

Rita Dreier

Yifei Du

M. Beatriz Durán‐Alonso

Amit Dutt

Nejat Duzgunes

Piotr Dziegiel

Ivano Eberini

Sherien M. El‐Daly

Carlos A. Elena‐Real

Elis Eleutherio

Jorg Enderlein

Jason Eng

Sarah England

Phei Er Saw

Laura Evangelista

Paola Fabbri

Alan H. Fairlamb

Franco Falcone

Emilia Falcone

Minrui Fan

Laszlo Farkas

Candida Fasano

Carol Feghali‐Bostwick

Yansheng Feng

Ruofei Feng

Yingang Feng

Julian Fernandez

Belen Fernandez

Alejandro Ferrando

Samuele Ferrari

László Fésüs

Ana Lúcia Figueiredo Porto

Arnaud Firon

Rachel Floyd

David Foster

Sergio Eduardo Longo Fracalanzza

Hector Franco

Andrew Fry

Jing Fu

Zhongjie Fu

Naonobu Fujita

Yasuyuki Fujiwara

Yoshitaka Fukada

Atsunori Fukuhara

Helena Fulka

Tadaomi Furuta

Shinya Fushinobu

Hans‐Joachim Gabius

Shrikanth Gadad

Jennifer Gamble

Mariana García

Irma Garcia Martinez

José Manuel García‐Heredia

James A. Garnett

Domenico Garozzo

Marie‐Line Garron

Maria Gazouli

Elena Gazzano

Róna Gergely

Reena Ghildyal

Alessandro Giuffrè

Howard Goldfine

Jorge Enrique Gómez‐Marín

Pablo Gonzalez

Aliesha Gonzalez‐Arenas

Katiuska González‐Arzola

Mark D. Gorrell

Sergio A. Gradilone

Barish Grant

Julie Grantham

Darrell Green

Jonathan Green

Elvira V. Grigorieva

Ramon Grima

Valentina Grossi

Reinhard Gruber

Henri Gruffat

Paolo Grumati

Wenchao Gu

Yifeng Gu

Pablo Guardado‐Calvo

Alejandra Guerra‐Castellano

Nancy Guillen

Siqi Guo

Ishaan Gupta

Emilio Gutierrez

Emma E. Hamilton‐Williams

Marshall Hampton

Shuping Han

Anis Hanna

Nan Hao

Richard P. Harbottle

Anandwardhan A. Hardikar

Brittney Harrington

Tarique Noorul Hasan

Kazuhiko Hashimoto

Jeffrey S. Haug

Lukas J. A. C. Hawinkels

Yi He

Ines Heiland

Udo Heinemann

Wiljan Hendriks

Michael J. Hendzel

Rob Henning

R. Henrique

Shane Hickey

Andrew Higham

Masaki Hiramoto

Kazumi Hirano

Kouji Hirota

Kohji Hizume

Tie Hong

John D. Hooper

Sandrine Horman

Ming‐Hon Hou

Guy A. Howard

Tsung Hua Hsieh

Chia‐Ling Hsieh

Jia Hu

Yongye Huang

Danny Huang

Gloria Huerta‐Ángeles

Matthias Husmann

Maik Hüttemann

Taisen Iguchi

Maria Ikonomopoulou

Andrea Ilari

Cristhian Ildefonso

Didem Ilter

Takeshi Imai

Anne Imberty

Nobuya Inagaki

Valentina Indio

Jin‐ichi Inokuchi

Hideki Inoue

Yuji Isegawa

Claudio Isella

Rie Ishikawa

Takeshi Ito

Nina Ivanovska

Tomoyuki Iwata

Elisa Helena Farias Jandrey

Jean‐Michel Jault

Jolanta Jaworek

Seon‐Yong Jeong

Peihua Jiang

Sonia Jimeno

Silvia Jimeno‐González

Cheng‐Hao Jin

Marek Jindra

Philip E. Johnson

Christopher N. Johnson

Shannon Joseph

Ivana Josipovic

Muhammad Junaid

Klaus Jung

Dieter Kabelitz

Pamela Kakimoto

Apinun Kanpiengjai

Boleslaw Karwowski

Muzaffer Ahmad Kassab

Takahiro Kato

Pravin Kaumaya

Gurmeet Kaur

Seiji Kawamoto

Koichiro Kawashima

Younghoon Kee

Lindsey Kennedy

Ian D. Kerr

Sung Hwan Ki

Sunhong Kim

Sung Hyun Kim

Yoko Kimura

Uday Kishore

Chandra Kishore

Jeffery Klauda

Alexa Karina Klettner

Dagmara Kłopotowska

Erika L. Knott

Thilo Kohler

Masato Koike

Naoya Kojima

Kensei Komatsu

Hiroyuki Konishi

Oliver H. Krämer

Jeffrey Kroon

Takashi Kudo

Veerapol Kukongviriyapun

Peter Istvan Kulcsar

Sandeep Kumar

Dhiraj Kumar

Sachin Kumar

Mukesh Kumar

Ashok Kumar

Toshiaki Kume

Rita Kundu

Petri Kursula

Ozgur Kutuk

Lawrence N. Kwong

Hirohisa Kyogoku

Leticia Labriola

Csilla Laczka

Timothy Y. Y. Lai

Sowmya Lakshmi

Gilles Lalmanach

Mauld Lamarque

Otmane Lamrabet

Olga Lancho

João Laranjinha

H. Peter Larsson

Janine M. LaSalle

Gregory Lavieu

Steven James Lawrence

Maciej Lech

S.Y. Lee

Tzong‐Shyuan Lee

Jin‐A Lee

Roger Leng

Martina Lepore Signorile

Gaetano Leto

François Letourneur

Shugang Li

Xaria Li

Xiwen Li

Jane B. Lian

Florence Lichou

Daniela Liebsch

Ya‐Wen Lin

Zoltan Lipinszki

Mónika Lippai

Zheng‐Zhao Liu

Ganbg Liu

Jian Liu

Yan‐Qun Liu

Yuchen Liu

Xingguo Liu

Sivakumar Loganathan

Giselle Lopes

Haya Lorberboum‐Galski

Ricardo O. Louro

Thomas Loustau

Qingchun Lu

Lingeng Lu

Edlira Luca

Shukun Luo

Sihui Ma

Yongjie Ma

Emilia Maellaro

Andrea Magrì

Bibekanand Mallick

Balca Mardin

Luis Antonio Mariñas‐Pardo

Mozart Marins

Sylvie Marleau

Sergio Marti

Jonathan Martinez‐Fabregas

Emanuela Masini

Pedro Matias

Masatoshi Matsunami

Yoshiyuki Matsuo

Ryo Matsuura

Aránzazu Mediero

Muthamilarasan Mehanathan

Thomas Melendy

Carlos Frederick Martins Menck

Nerea Mendez‐Barbero

Yssel Mendoza‐Marí

Tamas Meszaros

Frank Michelangeli

Anna Maria Mileo

Akira Mima

Junsei Mimura

Jonathan Minden

John D. Minna

Joana P. Miranda

Hamed Mirzaei

Nico Mitro

Kosuke Mizutani

Miguel Mompeán

Luca Monticelli

Leire Moreno

Katsutaru Morino

Assaf Mosquna

Jorge Mukdsi

Shona Murphy

Kevin Myant

Taku Nagai

Athi Naganathan

Shruti Nagaraja

Shuichi Nakamura

Ikuhiko Nakase

Minas Nalbandian

Heinz Peter Nasheuer

Viswanathan Natarajan

Ophir Nave

Alex V. Nechiporuk

Hovav Nechushtan

Simon Newstead

Tan Nguyen

Juan Nicola

Henrietta M. Nielsen

Kacper Nijakowski

Sasha Nikolic

Keisuke Nimura

Hiroyuki Nojima

Hiroshi Nomura

Mohsen Norouzinia

Cristina Nowicki

Yong‐Seok Oh

Valentyn Oksenych

Mateusz Olbromski

Érica Aparecida de Oliveira

Fabiola Olivieri

Tuğba Önal‐Süzek

Robert Opitz

Stjepan Orhanović

Edgardo Ortiz

Nigel Page

Jinghua Pan

Chinmay Kumar Panda

Agnieszka Paradowska‐Gorycka

Bhavin Parekh

Arli Aditya Parikesit

Inkyu Park

William Parson

Rakesh Pravinchandra Patel

Andrej Pavlovic

Milena Paw

Stevan Pecic

Petr Pecina

Gustavo Pedraza‐Alva

Osvaldo Rodrigues Pereira Junior

Oscar Perez‐Mendez

Rosario Perona

Shazib Pervaiz

Alessia Peserico

A. Petry

Bruce Pfeffer

Karolina Pierzynowska

Giuseppe Pignataro

Adrian Pinto‐Tomas

Astrid Pinzano

Karolina Pircs

Wojciech Plazinski

Emmi Poikulainen

Indira D. Pokkunuri

Baruh Polis

Raffaele Porta

Lourival Domingos Possani

Sean Post

Robert Potts

Kontantinos Poulas

David R. Poyner

Vasilis J. Promponas

Anna Przybylska‐Piech

Glen Pyle

Zhengtang Qi

Hui‐Min Qin

Zhenghong Qin

Runze Quan

Thierry Rabilloud

Stefania Raimondo

Malini Rajan

Peramaiyan Rajendran

Sriganesh Ramachandra Rao

Joao Ramalho‐Santos

Eric Gustavo Ramírez‐Salazar

Sonia Ramos

Minakshi Rana

Mohammad Reza Raoufy

Alberto Rascon

P. Hemachandra Reddy

Divijendra Natha Reddy S.

Simona Reina

Thomas Reubold

N. Rhaleb

Claudia Ribeiro

Sonia Rocha

Karin Rodland

Cesar Rodriguez

Jelena Roganovic

Katarzyna Rolle

Sandra Romero‐Cordoba

Yikang S. Rong

Georcki Ropón‐Palacios

Kyle Roux

Adhiraj Roy

Yuri Rubtsov

Donald Ruhl

Gian Luigi Russo

Seongho Ryu

Maja Sabol

Martin Kwasi Safo

Girish Sahni

Gloria Salazar

Ali Salehzadeh‐Yazdi

Mario Salvi

Mauro Salvi

Paola Sanese

Prasanna Kumar Santhekadur

Thiago de Santana Santos

Gerardo Santos‐López

Dhifaf Sarhan

Yasushi Sasaki

Naoyuki Sato

Shingo Sato

Pratik Satya

Pauline Schaap

Martina Schifferer

Thomas Schmidt

Barbara Schnierle

Kristin Schubert

Christine Schwartz

John Seddon

Rony Segers

Yusuke Sekine

David Selva

Iurii Semenov

Ivana Semova

Juan Carlos Sepúlveda‐Arias

Qingli Shang

Bojing Shao

Kunal Sharan

Jyoti Sharma

Zheyu Shen

Masakazu Shinohara

Takahiro Shiotsuki

Keiro Shirotani

Varda Shoshan‐Barmatz

Tsuyoshi Shuto

Mohammad Imran Siddiqi

Edmund Ui‐Hang Sim

Purva Singh

Richa Singhania

Lukasz Skalniak

Chad Slawson

Agnieszka Śmieszek

Ivan Smirnov

David Smith

Martyn T. Smith

Akira Sobue

Felix Sommer

Myungsun Son

Yang Song

Minseok Song

Rana Soylu‐Kucharz

Brett T. Spear

Pietro Speziale

L. V. Spirina

Anje Sporbert

Piyarat Srisawang

Ashok Srivastava

Alexey Stepanov

Angela Stoddart

Frank Stubenrauch

Chongyu Su

Abdulhamit Subasi

Chiara Succi

Chang Sun

Zhen Sun

Yuan Sun

Prashanth N. Suravajhala

Burcu Biterge Sut

Youri Svitkin

Aron Szabo

Yasuhiko Tabata

Ehsan Taghiabadi

Ryosuke Takahashi

Tetsufumi Takahashi

Shunichi Takeda

Hisanori Takenobu

Jun Takeuchi

Iman Talaat

Francesco Tamagnini

Markku Ilmari Tammi

Xiaojun Tan

Xiao‐Han Tang

Fuzhou Tang

Jason Tanny

Muhammed Bahattin Tanyolac

Umberto Tarantino

Seong Lin Teoh

John Termini

Suman S. Thakur

Rajesh K. Thimmulappa

Vengatesen Thiyagarajan

Gabor Joseph Tigyi

Vjekoslav Tomaic

Maria Lauda Tomasi

Takehiko Tosha

Déborah Tribouillard‐Tanvier

Chakrapani Tripathi

Timir Tripathi

Takashi Tsuboi

Kouhei Tsumoto

Hannele Tuominen

Paola Turano

Shusaku Uchida

Takeshi Uchida

Wanvisa Udomsinprasert

Jesús Ureña

Taro Uyeda

Tomas Vacik

Nadine van Montfoort

Zoltan Varga

Basavaraj Vastrad

Chanabasayya Vastrad

Alejandro Velazquez‐Cuz

Andrea Vergara

Joaquim Vives

Susanne Voelkel

Benjamin Voellger

Ivan I. Vorobiev

Gerassimos E. Voutsinas

Darcy Wagner

Stephen J. Walker

Verena Wally

Stephan Walrand

Zhichao Wang

Weili Wang

Feng Wang

Ye Wang

Yunguan Wang

Kun Wang

Yin Wang

Jun Wang

Xiaoding Wang

Matthew J. Wargo

Helen Watson

Jean‐Luc Wautier

Dong‐Qing Wei

Neale Weitzmann

Tim J. M. Welting

Johannes Westman

Judith M. White

Theresa Whiteside

Joanna Wietrzyk

Anne Willis

Stephen G. Withers

Kenneth W. Witwer

Remigiusz Worch

Ming‐Jiuan Wu

Yuan Seng Wu

Nan Wu

Xue Xiao

Junbo Xie

Bing‐Cong Xing

Yibin Xu

Shi‐Qing Xu

Jiaping Xu

Ming Xue

Tomio Yabe

Sudhir Kumar Yadav

Yasuyuki Yamada

Atsushi Yamamoto

Toshinari Yamasaki

Tomohiro Yamashita

Biao Yan

Jian Yan

Yungui Yang

Zhaolin Yang

Xi Yang

Yueh‐Hsun Kevin Yang

Masafumi Yano

Chi‐Tai Yeh

Haidi Yin

Jari Ylanne

Do‐Young Yoon

Shinya Yoshikawa

Seock‐Won Youn

Qiangfeng Yu

Xiaojie Yu

Ziniu Yu

Louis Yuge

Mone Zaidi

Charlotte U. Zajc

Fabiana Fonseca Zanoelo

Liang Zhang

Jian Zhang

Fang‐Jie Zhang

Yong Zhang

Jinfa Zhang

Liangqing Zhang

Jing‐Pu Zhang

Lingqiang Zhang

Qingyang Zhang

Yunfeng Zhao

Shushan Zhao

Baobing Zhao

Peng Zhao

Yun‐Wen Zheng

Joy Zhou

Huiping Zhou

Yanfei Zhou

Angela Ziebell

Janja Zupan

